# Characterizing the interindividual postexercise hypotension response for two order groups of concurrent training in patients with morbid obesity

**DOI:** 10.3389/fphys.2022.913645

**Published:** 2022-10-10

**Authors:** Cristian Álvarez, Francisco Guede-Rojas, Rodrigo Ramírez-Campillo, David C. Andrade, Jaime Vásquez-Gómez, Fernando Rodríguez-Rodríguez, Emmanuel Gomes Ciolac, Felipe Caamaño-Navarrete, Pedro Delgado-Floody

**Affiliations:** ^1^ Exercise and Rehabilitation Sciences Institute, School of Physical Therapy, Faculty of Rehabilitation Sciences, Universidad Andres Bello, Santiago, Chile; ^2^ Exercise Applied Physiology Laboratory, Centro de Investigación en Fisiología y Medicina de Altura, Departamento Biomedico, Facultad de Ciencias de la Salud, Universidad de Antofagasta, Antofagasta, Chile; ^3^ Centro de Investigación de Estudios Avanzados del Maule (CIEAM), Laboratorio de Rendimiento Humano, Universidad Católica del Maule, Talca, Chile; ^4^ IRyS Group, Physical Education School, Pontificia Universidad Católica de Valparaiso, Valparaíso, Chile; ^5^ Exercise and Chronic Disease Research Laboratory, Department of Physical Education, School of Sciences, Sáo Paulo State University (UNESP), Bauru, Buazil; ^6^ Physical Education Career, Universidad Autónoma de Chile, Temuco, Chile; ^7^ Department of Physical Education, Sport and Recreation, Universidad de La Frontera, Temuco, Chile; ^8^ Department Physical Education and Sports, Faculty of Sport Sciences, University of Granada, Granada, Spain; ^9^ Strength & Conditioning Laboratory, CTS-642 Research Group, Department Physical Education and Sports, Faculty of Sport Sciences, University of Granada, Granada, Spain

**Keywords:** exercise, concurrent training, endurance training, obesity, morbid obesity, nonresponders, blood pressure, metabolic syndrome

## Abstract

**Background:** Postexercise hypotension (PEH) is a common physiological phenomenon occurring immediately after endurance training (ET), resistance training (RT), and ET plus RT, also termed concurrent training (CT); however, there is little knowledge about the interindividual and magnitude response of PEH in morbidly obese patients.

**Aim:** The aims of this study were (1) to investigate the effect of CT order (ET + RT vs. RT + ET) on the blood pressure responses; 2) characterize these responses in responders and nonresponders, and 3) identify potential baseline outcomes for predicting blood pressure decreases as responders.

**Methods:** A quasi-experimental study developed in sedentary morbidly obese men and women (age 43.6 ± 11.3 years; body mass index [BMI] ≥40 kg/m^2^) was assigned to a CT group of ET plus RT (ET + RT; *n* = 19; BMI 47.8 ± 16.7) or RT plus ET order group (RT + ET; *n* = 17; BMI 43.0 ± 8.0). Subjects of both groups received eight exercise sessions over four weeks. Primary outcomes include systolic (SBP), diastolic (DBP), mean arterial pressure [MAP], heart rate at rest [HR], and pulse pressure [PP] measurements before and after 10 min post-exercise. Secondary outcomes were other anthropometric, body composition, metabolic, and physical fitness parameters. Using the delta ∆SBP reduction, quartile categorization (Q) in “high” (Rs: quartile 4), “moderate” (MRs: quartile 3), “low” (LRs: quartile 2), and “nonresponders” (NRs: quartile 1) was reported.

**Results:** Significant pre–post changes were observed in ET + RT in session 2 for SBP (131.6 vs. 123.4 mmHg, *p* = 0.050) and session 4 (131.1 vs. 125.2 mmHg, *p* = 0.0002), while the RT + ET group showed significant reductions in session 4 (134.2 vs. 125.3 mmHg, *p* < 0.001). No significant differences were detected in the sum of the eight sessions for SBP (∑∆SBP) between ET + RT vs. RT + ET (−5.7 vs. −4.3 mmHg, *p* = 0.552). Interindividual analyses revealed significant differences among frequencies comparing Q1 “NRs” (*n* = 8; 22.2%), Q2 “LRs” (*n* = 8; 22.2%), Q3 “MRs” (*n* = 9; 25.0%), and Q4 “HRs” (*n* = 11; 30.5%), *p* < 0.0001. Quartile comparisons showed significant differences in SBP changes (*p* = 0.035). Linear regression analyses revealed significant association between ∑∆SBP with body fat % (β –3.826, *R* ^2^ 0.211 [21.1%], *p* = 0.031), skeletal muscle mass [β –2.150, *R* ^2^ 0.125 (12.5%), *p* = 0.023], fasting glucose [β 1.273, *R* ^2^ 0.078 (7.8%), *p* = 0.003], triglycerides [β 0.210, *R* ^2^ 0.014 (1.4%), *p* = 0.008], and the 6-min walking test [β 0.183, *R* ^2^ 0.038 (3.8%), *p* = 0.044].

**Conclusion:** The CT order of ET + RT and RT + ET promote a similar ‘magnitude’ in the postexercise hypotensive effects during the eight sessions of both CT orders in 4 weeks of training duration, revealing “nonresponders” and ‘high’ responders that can be predicted from body composition, metabolic, and physical fitness outcomes.

## Introduction

Physical inactivity [i.e., not adhering to international physical activity guidelines of a minimum of 150 min of physical activity of moderate intensity, or 75 min of vigorous physical activity/week (Kanaley et al., 2022), sedentary behavior, together with other lifestyle conditions (i.e., unhealthy nutrition or poor sleep time) promote obesity, and cardiometabolic diseases such as type 2 diabetes mellitus (T2DM) and arterial hypertension (HTN) (Booth et al., 2012), two major diseases in the modernity, are of high prevalence in populations with excessive adiposity, such as those with morbid obesity (Booth et al., 2012).

Unfortunately, metabolic syndrome (MetS), another complex cardiometabolic square that also implies obesity, poor glucose control, high blood pressure, dyslipidemia, and high triglycerides in plasma, exacerbates the risk for suffering from cardiovascular diseases, particularly to those with morbid obesity who are candidates for bariatric surgery (Bellicha et al., 2021). For example, a relevant meta-analysis of 87 studies with a sample of (*n* = 951.083) showed that MetS risk factors were associated with an increased risk for cardiovascular disease (CVD) [relative risk, (RR) = 2.35], CVD mortality (RR = 2.40), myocardial infarction (RR = 1.99, stroke (RR = 2.27), and all-cause mortality (RR = 1.58) (Mottillo et al., 2010). Patients with morbid obesity are a particular population that suffers from multiple risks for CVD, including MetS, endothelial dysfunction, HTN and T2DM risk factors, all related to a better or worse profile to deal with success and better recovery period after bariatric surgery (Kanaley et al., 2022). From the National Health Survey of Chile, it was discovered that morbid obesity is a worrying concern in women of childbearing ages, 20 to 45 years, showing almost no changes from 2003 (2.2%) to 2009–10 survey (2.1%) (Araya et al., 2014). Thus, among these patients, to acquire the bariatric surgery benefit increase their possibilities to lose excess of weight by close to ∼50%, and thus to recover their overall cardiometabolic health (Deitel and Shahi, 1992), being this strongly related with improvements in their quality of life (Sierżantowicz et al., 2022).

Henceforth, lifestyle interventions, such as exercise training, play a key role (Flück, 2006; Floody et al., 2015; Delgado-Floody et al., 2020), and relevant international organizations such as the American College of Sports Medicine (Kanaley et al., 2022) and the European Association For The Study of Obesity Physical Activity Working Group (Oppert et al., 2021) have recently highlighted their benefits before and after bariatric surgery. Exercise training is a known but poorly used strategy for controlling cardiometabolic risk in populations with T2DM, HTN, and MetS (Booth et al., 2002; Flück, 2006). At least in the blood pressure and vascular context, exercise training decreases chronic and acute blood pressure levels (Cornelissen and Fagard, 2005; Álvarez et al., 2013; Bonsu and Terblanche, 2016; Cano-Montoya et al., 2021). For example, a single session of endurance training (ET) (also known as moderate-intensity continuous training) decreased the 24-h blood pressure (Karoline De Morais et al., 2015), and the magnitude of this effect was superior in subjects with higher blood pressure (systolic [SBP]/diastolic [DBP] −16.1/−7.5 mmHg) (Karoline De Morais et al., 2015) than in those with HTN controlled and HTN not so controlled (Ciolac et al., 2008), but the effect was clearly less marked in healthy normotensive subjects (SBP/DBP −5.6/−3.1 mmHg) (Park et al., 2006). These effects have been confirmed by a relevant meta-analysis where long-term ET has been a recognized strategy to decrease blood pressure in pre- and hypertensive populations (Cornelissen and Smart, 2013; Montero et al., 2014). On the other hand, resistance training (RT) have been also demonstrated to decrease blood pressure, for example by only 45 min after an acute RT session in HTN subjects (SBP/DBP −22.0/−8.0 mmHg), and this has also been previously confirmed by a meta-analyses which summarized these blood pressure decreases after RT in −4.4 mmHg (Kelley and Kelley, 2000). Recently, and in similar coherence with ET, the RT modality has reported a higher magnitude of BP reduction in subjects with elevated (SBP/DBP −12.0/−4.0 mmHg) than in healthy subjects (SBP/DBP −4.0/−1.0 mmHg) (Farinatti et al., 2021). Henceforth, the postexercise hypotension (PEH) [i.e., defined as a reduction in SBP/DBP arterial blood below control levels after a single bout of exercise (Kenney and Seals, 1993)] is the first vascular benefit immediately after acute exercise to acquire meaningful relevance, in terms that the summary of physiological adaptations by each exercise session can promote a renewal of the vasculature structure using molecular mechanisms (La Favor et al., 2016). Thus, as after both ET and RT, there are a number of evidences reporting the PEH benefits in different cohorts, but unfortunately, there is little evidence reporting this phenomenon after concurrent training (CT), and yet less knowledge in populations with MetS risk factors, as those with morbid obesity.

On the other hand, the main PEH studies report data in terms of ‘average data’, and it is well-known that there is a wide interindividual variability to exercise training (Bouchard and Rankinen, 2001), having a need for the report of data at an individual level. Thus, considering that there is little knowledge about the role of the order (i.e., starting by ET plus RT or by RT plus ET) in the CT session, taking into account that risk factors for MetS increase the risk for vascular damage and CVD, the aims of this study were (1) to investigate the effect of CT order (ET + RT vs. RT + ET) in the blood pressure responses, 2) characterize these responses in responders and nonresponders, and 3) identify potential baseline outcomes for predicting blood pressure decreases as responders. We hypothesized that, independently of the “magnitude” in the blood pressure changes, both CT order promotes blood pressure decreases, where similar to other populations, some subjects can be more Rs than other peers, and where some baseline anthropometric/body composition and metabolic or physical condition can be potential ‘predictors’ of this PEH response.

## Material and methods

### Subjects

#### Study design

This was a quasi-experimental study developed in patients with morbid obesity. The patients were invited for participating by a public invitation and directed to the Morbidly Obesity Association of Temuco, City, Chile (OBEMOB). After providing detailed information and feedback about the risks/benefits of the intervention, all participants signed an informed consent for participating in the study. We recruited diagnosed morbid obesity sedentary/physically inactive subjects (body mass index [BMI] between ≥40 kg/m^2^; aged 30–55 years) who were assigned for convenience in 1:1 allocation to a CT group of ET plus RT (ET + RT; *n* = 19; BMI 47.8 ± 16.7, 1 man) or a RT plus ET group (RT + ET; *n* = 17; BMI 43.0 ± 8.0, 1 man). Patients were recruited in 2021 from the abovementioned non-governmental institution. All participants were part of the lifestyle program previous to the possibility of receiving the bariatric surgery benefit for this disease condition from the Health Ministry of Chile (MINSAL).

**FIGURE 1:**
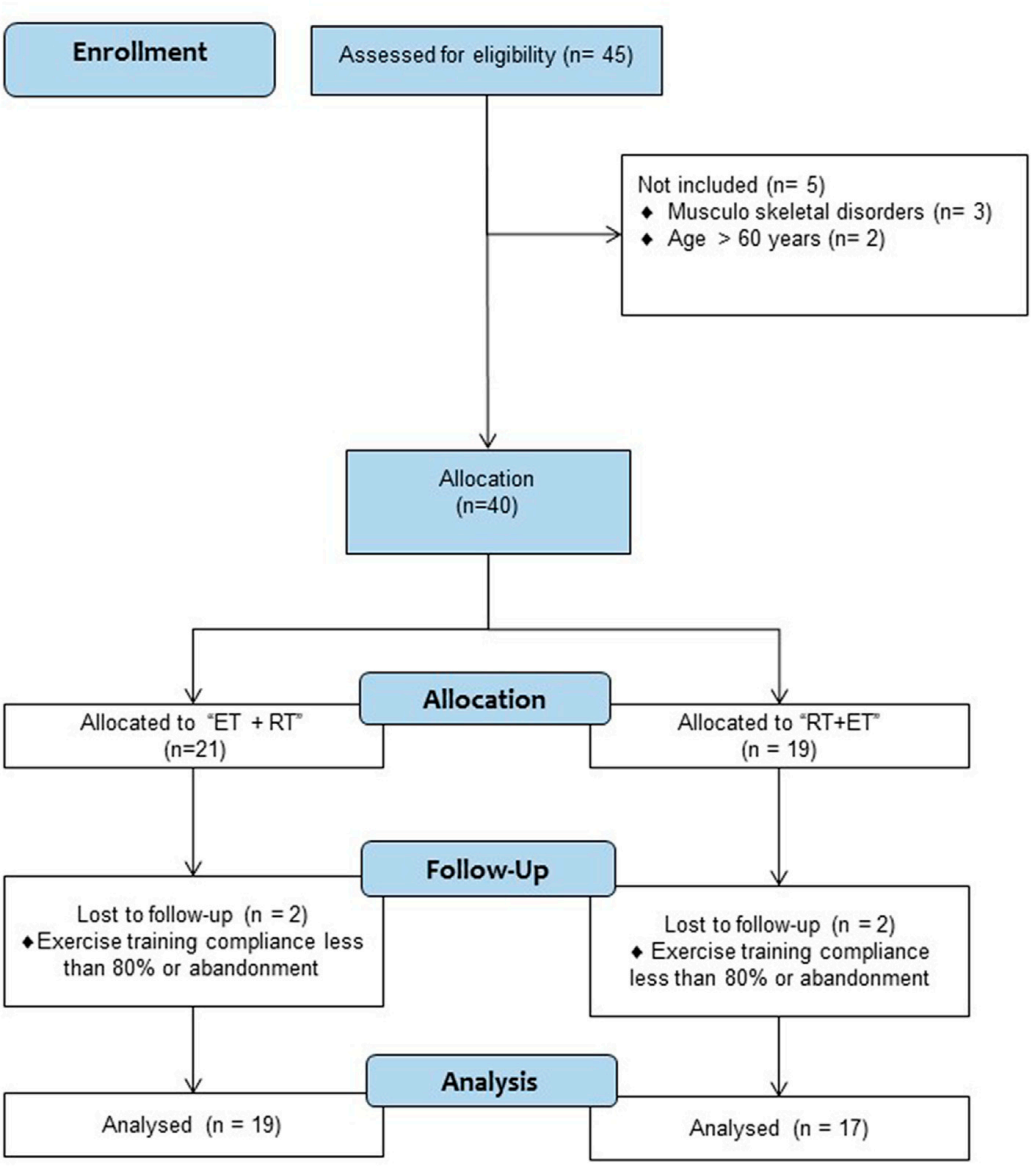
Study design.

The inclusion criteria were as follows: 1) age >18 and <60 years, 2) women or men, 3) medical authorization for physical tests, and 4) body mass index (BMI) ≥40 kg/m^2^ or between 35 and 40 kg/m^2^, but with previously diagnosed of morbid obesity. The exclusion criteria were as follows: 1) physical limitations to performing the physical test (e.g., restrictive injuries of the musculoskeletal system), 2) exercise-related dyspnea or respiratory alterations, and 3) chronic heart disease diagnosed during the time of morbid obesity diagnosed and the starting of the exercise intervention. In the enrolment stage, (*n* = 40) participants showed their intention for participating, being allocated to (ET + RT, *n* = 21, and RT + ET, *n* = 19). However, after the screening of inclusion/exclusion criteria, only (*n* = 36) were monitored after eight sessions of intervention (during 4 weeks) of follow-up, with two participants being excluded from each [ET + RT (*n* = 2) and RT + ET (*n* = 2)] group due to exercise adherence of less than 80%. Thus, the final sample size was as follows: (ET + RT; *n* = 19; BMI 47.8 ± 16.7) and (RT + ET; *n* = 17; BMI 43.0 ± 8.0). The study was carried out following the Declaration of Helsinki (2013) and was approved by the Ethical Committee of the Universidad de La Frontera, Temuco, Chile (ACTA Nº 080_21). The trial registration was part of the ClinicalTrials.gov ID: NCT05504629. The study design is shown in Figure 1.

### Concurrent training order groups

Independent of the order (*i.e.,* ET + RT, or RT + ET), each CT session had two sections, namely, RT and ET, which were applied two days per week, during eight sessions (4 weeks), and where the blood pressure changes were measured only in four opportunities (i.e.*,* once a week in sessions 2, 4, 6, and 8). Thus, the CT included in each session had three stages: 1) callisthenics exercises, 2) main exercise (ET + RT or RT + ET), and 3) cool down exercises.

Before starting, the participants were involved in the maximum strength capacity estimated using a submaximal test based on Brzycki’s equation: weight [1.0278-(0.0278*repetitions)] that does not change based on the sex, age of the population, or the exercise modality that is performed (Mayhew et al., 2004). After this procedure, four familiarization sessions were applied, which consisted of the following: Session 1: “knowledge of all measurements,” “knowledge of exercise-machine manipulation,” and “instructions during the exercise program”; Session 2: “exercising during cycling,” “exercising with weights,” and “exercising with metal bars”; Session 3: applying a few of the exercises of RT in 2–3 sets to know the configuration of each exercise (i.e., ET and RT regimes itself); and Session 4: “applying the 50–70% of their CT program” corresponding to a normal session compound. In the “calisthenic stage,” each subject developed 10-min warm-up with continuous walking, joint mobility exercises, and flexibility exercises. In the “main part” of the session, and independent of the CT order group (ET + RT or RT + ET), the ET section consisted of continuous cycling using a magnetic resistance static bicycle (Oxford™ Fitness, model BE-2701, Chile) for 20 min. The intensity of the ET exercise was regulated through the previously used modified Borg scale from 1 to 10 rating of perceived exertion (Gillen et al., 2013), and the participants worked at a level between 5 and 7 points (i.e., moderate-intensity), considering the original 6 to 20 point scale (Borg, 1998), being the subjects controlled to not exceed this intensity restriction. On the other hand, the RT section included eight to 12 muscle strength exercises of circuit training with the following different muscle groups: 1) forearm, 2) knee flexors and extensors, 3) trunk, 4) chest, 5) shoulder elevators, 6) horizontal shoulder flexors, 7) extensors and, finally, 8) plantar flexors. These exercises were performed in one set in a continuous concentric/eccentric voluntary contraction as possible for 60 s [until perceiving a vigorous intensity at the final stage of this 60 s using six to 20 points of the (Borg, 1998) scale, followed by 60–90 s of passive recovery], where each participant was changed to another exercise modality into the circuit. The RT section was of 25 min in duration time. The study protocol is shown in Supplementary Figure S1.

### Anthropometric and body composition assessments

Body mass (kg) was measured using a digital bioimpedance scale (TANITA™, model 331, Tokyo, Japan). Height (m) was measured using a SECA™ stadiometer (model 214, Hamburg, Germany), with subjects in light clothes and without shoes. The BMI was calculated as the body weight divided by the square of the height (kg/m^2^). The BMI was determined to estimate the degree of obesity (kg/m^2^) using standard criteria for obesity and morbid obesity classification (Sturm, 2007). The waist circumference (WC) was assessed using a tape measure graduated in centimeters (Adult SECA™) at the upper hipbone and the top of the right iliac crest, with a flexible and metal tape in a horizontal plane around the abdomen at the level of the iliac crest. The tape was snug but did not compress the skin and was parallel to the floor. The measurement was made at the end of a normal expiration. The body composition measurement included body fat (%), body fat (kg), lean mass (kg), skeletal muscle mass (kg), total body water (%), bone mass (kg), and basal metabolic rate (kcal), which were measured using a digital bio-impedance scale (TANITA™, model 331, Tokyo, Japan). The characteristics of the participants can be seen from (Table 1).

**TABLE 1:** Baseline characteristics of the groups.

	Groups		
	ET + RT	RT + ET	ET + RT vs. RT + ET *p-*value, ES *d* =
(n =)	17	13	
Age (y)	43.8 ± 11.9	43.4 ± 10.7	*p* = 0.932; 0.00
Anthropometric			
Height (cm)	158.3 ± 7.2	157.4 ± 8.2	*p* = 0.750; 0.00
Body mass (kg)	109.2 ± 22.7	106.2 ± 19.6	*p* = 0.712; 0.04
Body mass index (kg/m^2^)	47.8 ± 16.7	43.0 ± 8.0	*p* = 0.433; 0.02
Waist circumference (cm)	116.4 ± 14.2	115.5 ± 12.4	*p* = 0.859; 0.00
Body composition			
Body fat (%)	48.0 ± 3.9	47.8 ± 6.1	*p* = 0.571; 0.01
Body fat (kg)	53.0 ± 14.7	50.8 ± 14.3	*p* = 0.648; 0.00
Lean mass (kg)	56.0 ± 9.0	55.4 ± 6.6	*p* = 0.839; 0.00
Skeletal muscle mass (kg)	53.2 ± 8.5	52.6 ± 6.3	*p* = 0.839; 0.00
Bone mass (kg)	2.8 ± 0.4	2.8 ± 0.3	*p* = 0.839; 0.00
Total body water (L)	41.2 ± 8.0	40.6 ± 5.6	*p* = 0.809; 0.00
BMR (kcal)	1778.0 ± 311.4	1750.0 ± 233.4	*p* = 0.783; 0.00
Cardiovascular			
Systolic BP (mmHg)	134 ± 17	125 ± 15	*p* = 0.119; 0.06
Diastolic BP (mmHg)	89 ± 10	84 ± 9	*p* = 0.194; 0.04
Heart rate rest (beats/min)	82 ± 10	81 ± 12	*p* = 0.792; 0.00
Pulse pressure (mmHg)			
Metabolic			
Diabetes			
Fasting glucose (mg/dl)	95.5 ± 11.6	89.8 ± 9.7	*p* = 0.388; 0.07
Dyslipidemia			
Total cholesterol (mg/dl)	170.3 ± 28.6	193.6 ± 24.7	*p* = 0.133; 0.14
LDL-c cholesterol (mg/dl)	100.5 ± 29.4	111.4 ± 20.0	*p* = 0.467; 0.03
HDL-c cholesterol (mg/dl)	48.3 ± 11.7	59.6 ± 18.8	*p* = 0.150; 0.13
Triglycerides (mg/dl)	140.7 ± 91.7	134.4 ± 41.3	*p* = 0.886; 0.00
Endurance performance			
6Mwt (m)	517.9 ± 171.2	484.7 ± 125.3	*p* = 0.515; 0.01
Strength performance			
Handgrip Nd (kg)	30.5 ± 11.8	28.7 ± 7.0	*p* = 0.577; 0.00
Handgrip D (kg)	29.9 ± 12.4	27.9 ± 7.5	*p* = 0.575; 0.00

Data are shown in mean and ± standard deviation. Groups are described as the (ET + RT) endurance training plus resistance training group and the (RT + ET) resistance training plus endurance training group. Outcomes are described as (BMR) basal metabolic rate, (LDL-c) low-density lipids, (HDL-c) high-density lipids, (6Mwt) six-minute walking test, (Nd) non dominant hand, and (D) dominant hand. (d) denotes Cohen d effect size.

### Cardiovascular health assessment

The heart rate at rest, systolic blood pressure (SBP), and diastolic blood pressure (DBP) was measured in the sitting position after 10 min of rest before and 10 min after each exercise session, similar to the procedure in previous studies (Cunha et al., 2017). When the second measurement was ≥5 mmHg in difference, we did not consider this and took a new measurement. Two recordings were made, and the mean of the measurements was used for statistical analysis using an OMRON™ digital electronic BP monitor (model HEM 7114, Chicago, IL, United States). Thus, in the case of PEH detection, we used the common and tested reliability method of (PEH = postexercise blood pressure—pre-exercise blood pressure) by (Fecchio et al., 2017). We recommended that caffeine, exercise, and smoking be avoided before measurement at least 90 min before evaluation and each exercise session. After that, we calculated the mean arterial pressure (MAP) and the pulse pressure (PP) using both SBP and DBP data to provide more information.

### Metabolic assessment

After overnight fasting of 10 ± 2 h, all patients underwent a baseline assessment (pre-test) between 08:00 and 9:00 in the morning. All participants arrived at the health center for blood sample extraction of 5 ml to determine the plasma outcomes; fasting plasma glucose (FPG), high-density lipoprotein cholesterol (HDL-c), triglycerides (Tg), cholesterol total (Tc), and low-density lipoprotein cholesterol (LDL-c) were included. These measurements were taken in an external private laboratory, being applied the protocol in the same conditions as in previous reports (Delgado-Floody et al., 2021).

### Endurance performance assessment

The day after the metabolic measurements, the physical condition of the participants in both groups was measured by endurance and muscle strength testing. First, a 6-minute walking test (6 Mwt) was used to estimate cardiorespiratory fitness. Thus, according to the distance in meters, each subject was classified with a correlated cardiorespiratory fitness by the maximum oxygen consumption following the categories; ‘very low (<5^th^ percentile)’, ‘low’ (5^th^–25^th^ percentile), “regular” (26^th^–50^th^ percentile), “good” (51^st^–75^th^ percentile), “excellent” (76^st^–95^th^ percentile), and “superior” (>95^th^ percentile) (Dourado et al., 2021). This information was used only to graduate the load intensity in watts that widely vary among participants of both groups. The test was performed in a closed space on a flat surface (30 m long), with two reflective cones placed at the ends to indicate the distance. During the test, participants were assisted with instructions from an exercise physiologist about the time of walking, tiredness, and exercise cessation.

### Strength performance assessment

Handgrip strength (HGS) was assessed using a digital dynamometer (BaselineTM Hydraulic Hand Dynamometers, United States), which has been used in previous studies. Two attempts were made, measuring each hand, and the best result from each was selected. As mentioned previously, the mean value obtained was taken as the total score and implemented according to similar procedures (Álvarez et al., 2017).

### Statistical analyses

Data are presented as the mean ± standard deviation (SD). Normality and homoscedasticity assumptions for all data were checked using the Shapiro–Wilk and Levene tests. Wilcoxon’s test was used for non-parametric data. After this, we performed 2-way ANOVA (group x time) to test SBP, DBP, MAP, HR, and PP outcomes in absolute values, where Sidac´s post hoc was applied to identify potential differences between ET + RT vs. RT + ET. Additionally, delta changes (∆) from pre–post were calculated to SBP, DBP, MAP, HR, and PP outcomes to test by the independent Student *t*-test differences between groups, where the non-parametric Wilcoxon test was optionally applied for non-parametric variables. The Cohen *d* effect size was obtained with threshold values at 0.20, 0.60, 1.2, and 2.0 for small, moderate, large, and very large effects, respectively (Hopkins et al., 2009). These analyses were carried out using the statistical software Graph Pad Prism 8.0 (Graph Pad Software, San Diego, CA, United States). After this, all results in ∆ changes of the sum of SBP ∑∆SBP were individualized and presented in quartile to be categorized in those participants as “nonresponders” (NRs; Q1 with SBP < –1 mmHg), “low” responders (LRs; Q2 with SBP –1 to –9.9 mmHg), “moderate” responders (MRs; Q3 with SBP −10 to −18 mmHg), and “high” responders (HRs; Q4 with SBP ≥ −18 mmHg) according with their blood pressure changes (i.e., particularly decreases) as have been previously reported (Green et al., 2014). Linear regression was applied in the backward mode for testing those potential body composition and metabolic and physical condition outcomes predictors of the PEH. The ∑∆SBP decreases an average of the sessions 1 and 2 of both ET + RT and RT + ET groups related to the PEH (i.e., decreases in SBP) was used as “dependent” outcome, while age, height, body mass, BMI, waist circumference, body fat in % and kg, lean mass, skeletal muscle mass, bone mass, total body water, BMR, SBP, DBP, HR at rest, pulse pressure, fasting glucose, total cholesterol, LDL-c, HDL-c cholesterol, triglycerides, 6Mwt, and handgrip muscle strength (dominant and non-dominant arm) were used as “independent” predictors. Statistical analyses for these procedures were applied using SPSS software version 28 (SPSS™ Inc. Chicago, Illinois, United States). The alpha level was fixed at (*p* < 0.05) for all tests of statistical significance.

## Results

### Baseline measurements

There were no baseline differences between groups in the anthropometric, body composition, cardiovascular, and metabolic and physical condition of co-variables assessed (Table 1).

### Changes in blood pressure (main outcomes) after acute ET + RT and RT + ET

**FIGURE 2:**
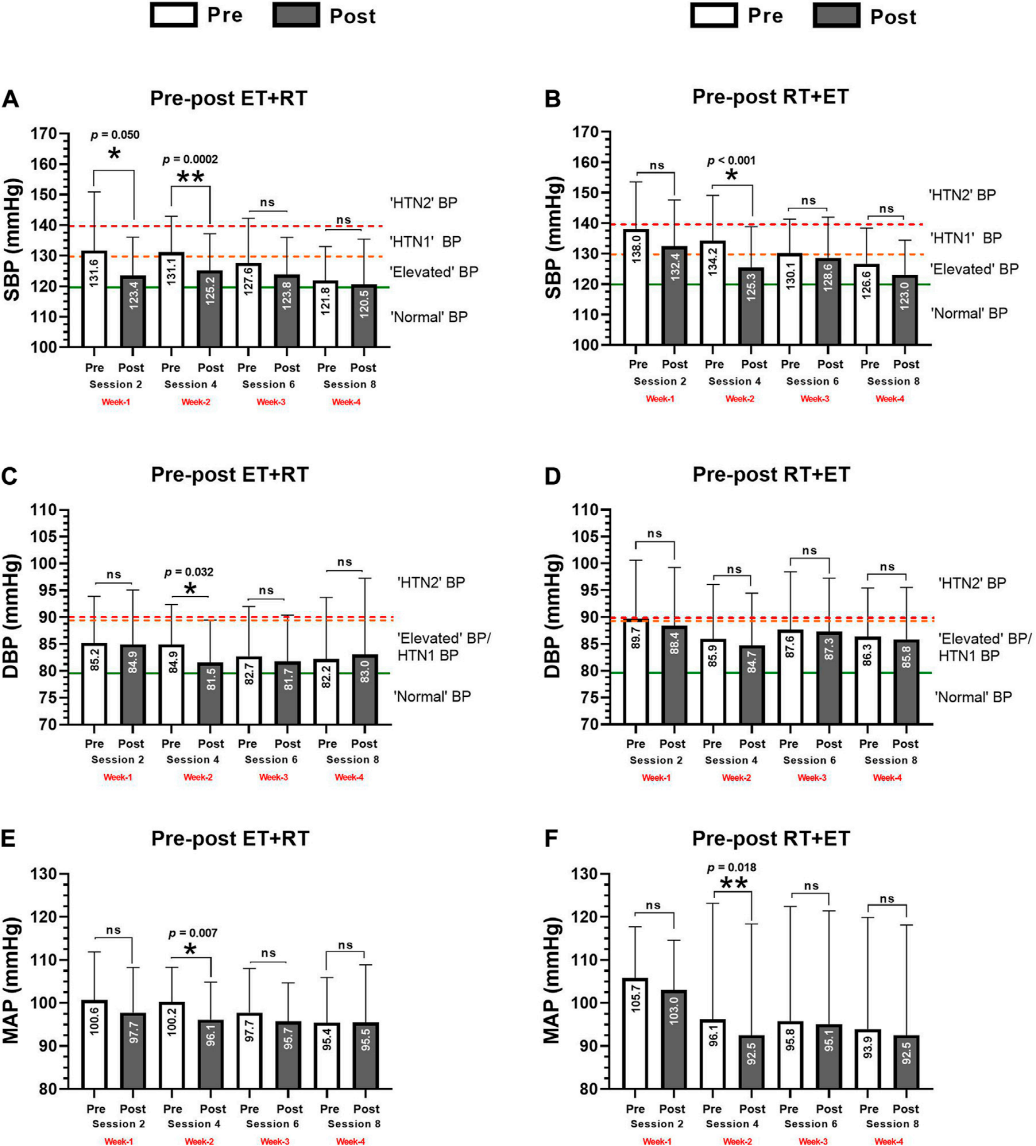
Acute absolute prepost changes in systolic **(A, B)**, diastolic **(C, D)**, and mean arterial blood pressure **(E, F)** after 4 weeks (eight exercise sessions) of two different orders of concurrent training in morbid obesity patients. Groups are described as (ET + RT) endurance training plus resistance training group and (RT + ET) resistance training plus endurance training group. Outcomes are described as (SBP) systolic, (DBP) diastolic, and (MAP) mean arterial pressure. Cut-off points of blood pressure are described as; “normal” BP, “elevated” BP, “HTN1” BP, and “HTN2” BP following standard classification criteria. The green continuous line denotes the limit for normal “normal” BP. The orange intermittent line denotes limit for “elevated” BP. The red intermittent line denotes the limit for “HTN2” BP. (*) denotes significant time prepost differences in each week at *p* < 0.05. (**) denotes significant time prepost differences in each week at *p* < 0.001. (ns) denotes no significant differences *p* > 0.05.

In the ET + RT group, there were significant reductions from pre- to post-test of SBP in the exercise session 1 (131.6 ± 19.1 to 123.4 ± 12.6, *p* = 0.050) and session 2 (131.1 ± 14.4 to 125.2 ± 11.9 mmHg, *p* = 0.0002) (Figure 2A). The RT + ET group showed significant reductions in SBP in session 2 (134.2 ± 14.8 to 125.3 ± 13.4, *p* < 0.001) (Figure 2B). In the ET + RT group, there were significant reductions in DBP in session 2 (84.9 ± 7.4 to 81.7 ± 7.8 mmHg, *p* = 0.032) (Figure 2C), while the RT + ET group do not elicited significant pre-post changes after each session (Figure 2D).). In MAP, the ET + RT group showed significant reductions in session 2 (100.2 ± 8.0 to 96.1 ± 8.7 mmHg, *p* = 0.007) (Figure 2E), and the RT + ET group showed similarly significant reductions in session 2 (96.1 ± 27.0 to 92.5 ± 25.8 mmHg, *p* = 0.018) (Figure 2F).

### Changes on heart rate and pulse pressure (secondary outcomes) after acute ET + RT and RT + ET

**FIGURE 3:**
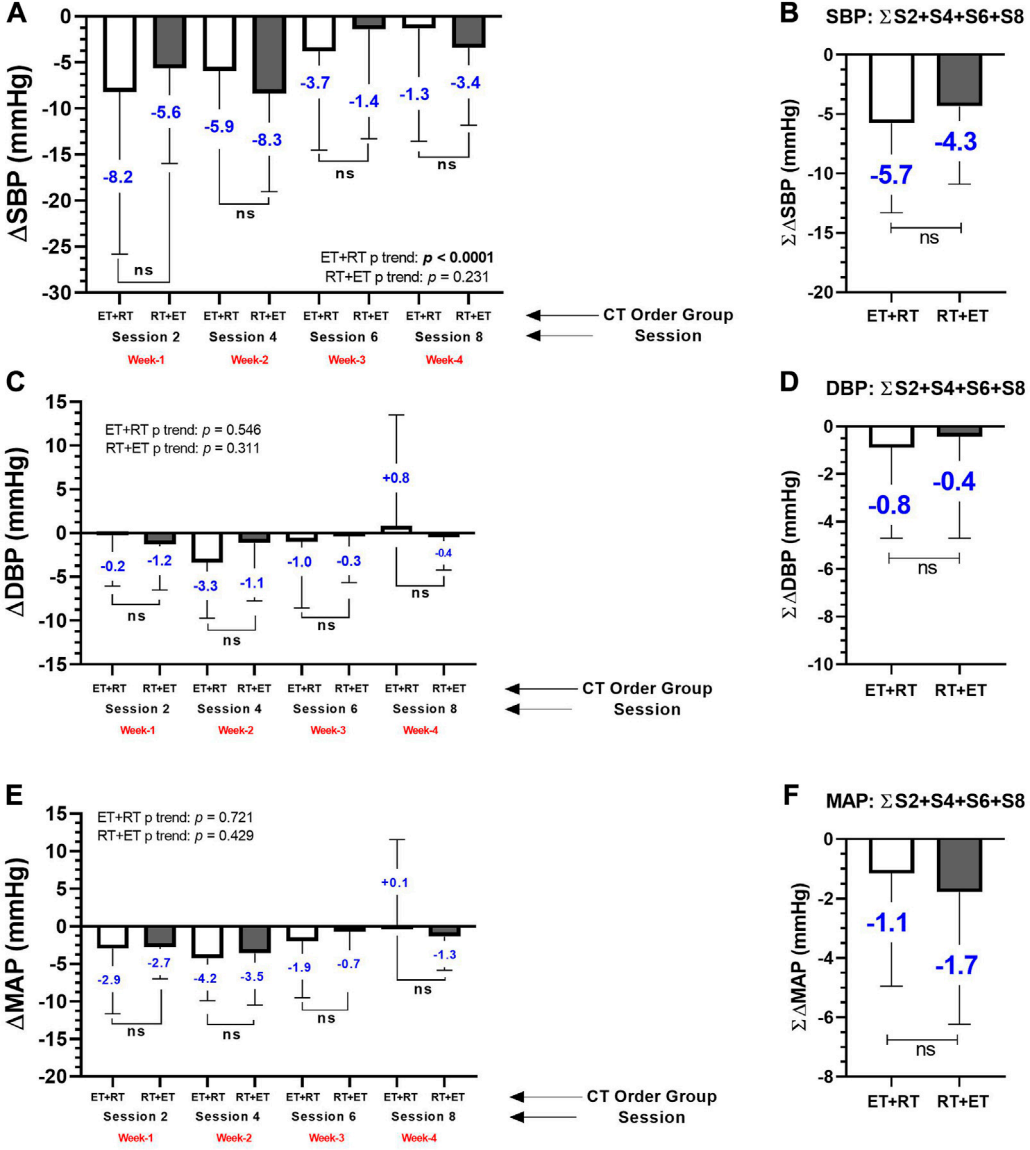
Postexercise hypotensive effect in systolic blood pressure by each delta changes by session **(A)**, and by the sum of 4 exercise sessions **(B)**, in diastolic blood pressure by each delta changes by session **(C)**, and by the sum of 4 exercise sessions **(D)**, and in mean arterial pressure by each delta changes by session **(E)**, and by the sum of 4 exercise sessions **(F)** during 4 weeks (eight exercise sessions) monitored four times during session 2 (second CT session), session 4 (fourth CT session), session 6 (sixth CT session), and session 8 (eight CT session) in morbid obesity patients’ participants of two order groups of concurrent training. Groups are described as (ET + RT) endurance training plus resistance training group and (RT + ET) resistance training plus endurance training group. Outcomes are described as (∆SBP) delta of systolic, (∆DBP) delta of diastolic, and (∆MAP) delta of mean arterial pressure. **(A)** ∆SBP decreases after four exercise sessions in each CT exercise group. **(B)**. The average (i.e., S2+S4+S6+S8) of these four measurements in each group, are abbreviated as ∑∆SBP **(B)**, being the same abbreviation as ∑∆DBP **(D)** and ∑∆MAP **(F)**. (ns) denotes no significant differences *p* > 0.05.

In the ET + RT group, there were significant increases in HR from pre- to post-exercise in session 1 (79.6 ± 12.5 to 89.7 ± 15.0, *p* < 0.0001), session 2 (81.0 ± 13.0 to 92.3 ± 12.5, *p* = 0.0002), session 3 (81.3 ± 12.5 to 91.0 ± 12.7, *p* < 0.0001), and post-exercise session 4 (78.8 ± 13.5 to 91.1 ± 10.0 beats/min, *p* = 0.0004) (Figure 3A). In the RT + ET group, there were significant increases from pre- to post-exercise in HR in session 1 (80.3 ± 11.8 to 94.2 ± 13.5, *p* = 0.0005), session 2 (78.6 ± 12.4 to 95.0 ± 11.0, *p* < 0.0001), session 3 (77.1 ± 12.6 to 92.9 ± 8.9, *p* < 0.0001), and post-exercise session 4 (83.9 ± 13.2 to 95.8 ± 12.1 beats/min, *p* < 0.0001) (Figure 3B). In the ET + RT group, there were significant decreases after the exercise session in PP of session 1 (46.4 ± 14.4 to 38.5 ± 7.6 mmHg, *p* = 0.020) (Figure 3C), and in the RT + ET group, there were significant decreases after exercise in PP of session 2 (45.4 ± 14.8 to 38.2 ± 12.6 mmHg, *p* < 0.001) (Figure 3D).

### “Magnitude” of the PEH in main and secondary outcomes by delta change comparisons between ET + RT and RT + ET

**FIGURE 4:**
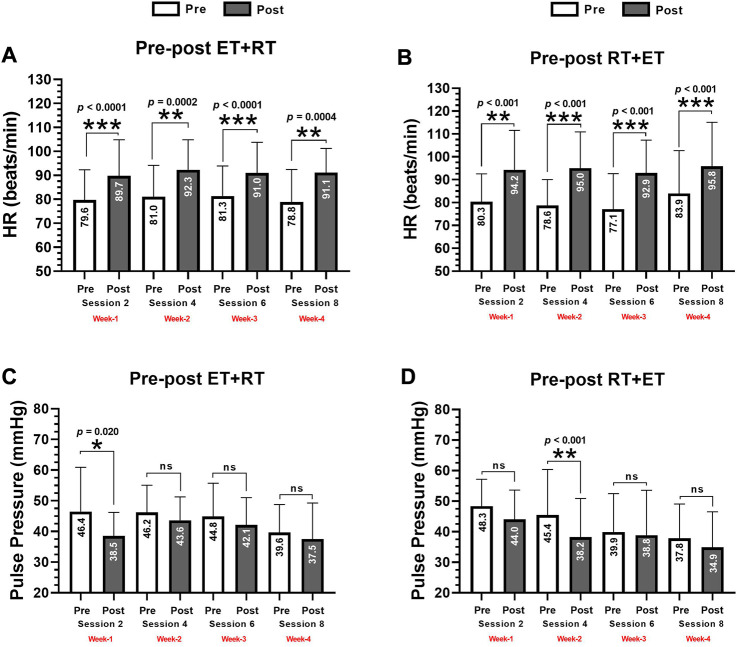
Acute absolute prepost changes in heart rate at rest **(A, B)** and in pulse pressure **(C, D)** after 4 weeks (eight exercise sessions) of two different orders of concurrent training in morbid obesity patients. Groups are described as (ET + RT) endurance training plus resistance training group and (RT + ET) resistance training plus endurance training group. Outcomes are described as (HR) Heart rate at rest. (*) denotes significant time prepost differences in each week at *p* < 0.05. (**) denotes significant time prepost differences in each week at *p* < 0.001. (***) denotes significant time prepost differences in each week at *p* < 0.0001. (ns) denotes no significant differences *p* > 0.05.

In the four sessions of measurements, there were different blood pressure decreases in the delta in both ET + RT and RT + ET groups at ∆SBP (session 2; −8.2 and −5.6 mmHg, session 4; −5.9 and −8.3, session 6; −3.7 and −1.4, and session 8; −1.3 and −3.4 mmHg), but no significant differences were observed comparing ET + RT vs. RT + ET in these four sessions (Figure 4A). There was a significant trend for a decline in the ‘magnitude’ of the ∆SBP decreases in the ET + RT group from sessions 2, 4, 6, and 8 (−8.8, −8.5, −2.1, and −1.1, *p* < 0.0001, respectively, to each session) (Figure 4A). Overall, considering the summation of the four exercise sessions (i.e., into 4 weeks) in ∆SBP (∑∆SBP), no significant differences were detected comparing ∑∆SBP of ET + RT vs. ∑∆SBP of RT + ET groups (−5.7 vs. −4.3 mmHg) (Figure 4B).

From pre- to post-exercise in each ET + RT vs. RT + ET group, there were different blood pressure decreases in both ET + RT and RT + ET at ∆DBP (session 2; −0.2 and −1.2, session 4; −3.3 and −1.1, session 6; −1.0 and −0.3, and session 8; +0.8 and −0.4 mmHg, respectively, to ET + RT and RT + ET), but comparing ∆DBP between ET + RT vs. RT + ET, no significant differences were detected in each session of measurement (i.e., session 2, 4, 6, and 8 of the blood pressure measurement) (Figure 4C). Overall, considering the summation of the four exercise sessions (i.e., into 4 weeks) (∑∆DBP), no significant differences were detected comparing ∑∆DBP of ET + RT vs. ∑∆DBP of RT + ET groups (−0.8 vs. −0.4 mmHg) (Figure 4D). At delta changes from pre to post each exercise session of measurement, there were different blood pressure changes in both ET + RT and RT + ET at ∆MAP (session 2; −0.9 and −2.7, session 4; −4.2 and −3.5, session 6; −1.9 and −0.7, and session 8; +1.0 and −.3 mmHg, respectively, to each ET + RT and RT + ET). However, no significant differences were observed comparing ET + RT vs. RT + ET in sessions 2, 4, 6, and 8 (Figure 4E). Overall, considering the summation of the four exercise sessions (*i.e*., into 4 weeks) (∑∆MAP), no significant differences were detected comparing ∑∆MAP of ET + RT vs. ∑∆MAP of RT + ET groups (−0.8 vs. −0.4 mmHg) (Figure 4F).

**FIGURE 5:**
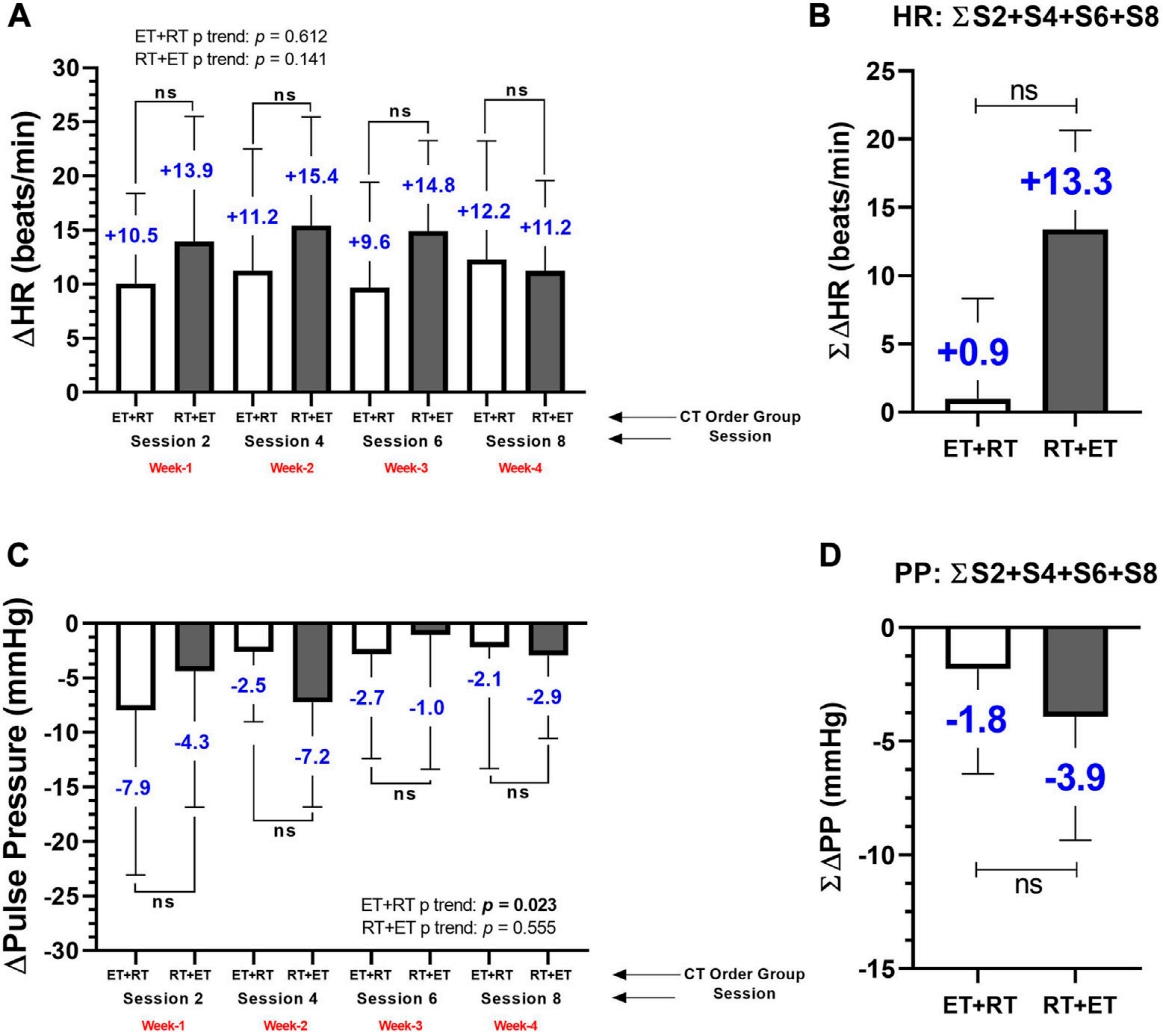
Acute delta changes in the heart rate at rest by each delta changes by session **(A)**, and by the sum of 4 exercise sessions **(B)**, and pulse pressure by each delta changes by session **(C)**, and by the sum of 4 exercise sessions **(D)** during 4 weeks (eight exercise sessions) monitored four times during session 2 (second CT session), session 4 (fourth CT session), session 6 (sixth CT session), and session 8 (eight CT session) in morbid obesity patients’ participants of two orders of concurrent training. Groups are described as (ET + RT) endurance training plus resistance training group and (RT + ET) resistance training plus endurance training group. Outcomes are described as (∆HR) delta of heart rate at rest **(A)**, and the average (i.e., S2+S4+S6+S8) of these four measurements in each group, are abbreviated as ∑∆HR **(B)**, (∆Pulse Pressure) delta changes of pulse pressure, and the average (i.e., S2+S4+S6+S8) of these four measurements in each group, are abbreviated as ∑∆PP **(D)**. (ns) denotes no significant differences *p* > 0.05.

From pre to post each exercise session of measurement, there were different delta changes in both ET + RT and RT + ET at ∆HR (session 2; +10.5 and +13.9, session 4; +11.2 and +15.4, session 6; +9.6 and +14.8, and session 8; +12.2 and 11.2 beats/min, respectively, to each ET + RT and RT + ET group), but no significant differences were detected comparing groups in each session 2, 4, 6, and 8 (Figure 5A). Overall, considering the summation of the four exercise sessions (i.e., into 4 weeks) (∑∆HR), no significant differences were detected comparing ∑∆HR of ET + RT vs. ∑∆HR of RT + ET groups (+0.9 vs. +13.3 beats/min) (Figure 5B). From pre to post each exercise session of measurement, there were different changes in both ET + RT and RT + ET at ∆PP (session 2; −7.9 and −4.3, session 4; −2.5 and −7.2, session 6; −2.7 and −1.0, and session 8; −2.1 and −2.9 mmHg, respectively, to each ET + RT and RT + ET group), but no significant differences comparing the ET + RT vs. RT + ET groups in each session 2, 4, 6, and 8 were detected (Figure 5C). Overall, considering the summation of the four exercise sessions (i.e., into 4 weeks) (∑∆PP), no significant differences were detected comparing ∑∆PP of ET + RT vs. ∑∆PP of RT + ET groups (−1.8 vs. –3.9 beats/min) (Figure 5D).

### Interindividual response to ET + RT and RT + ET order of concurrent training

**FIGURE 6:**
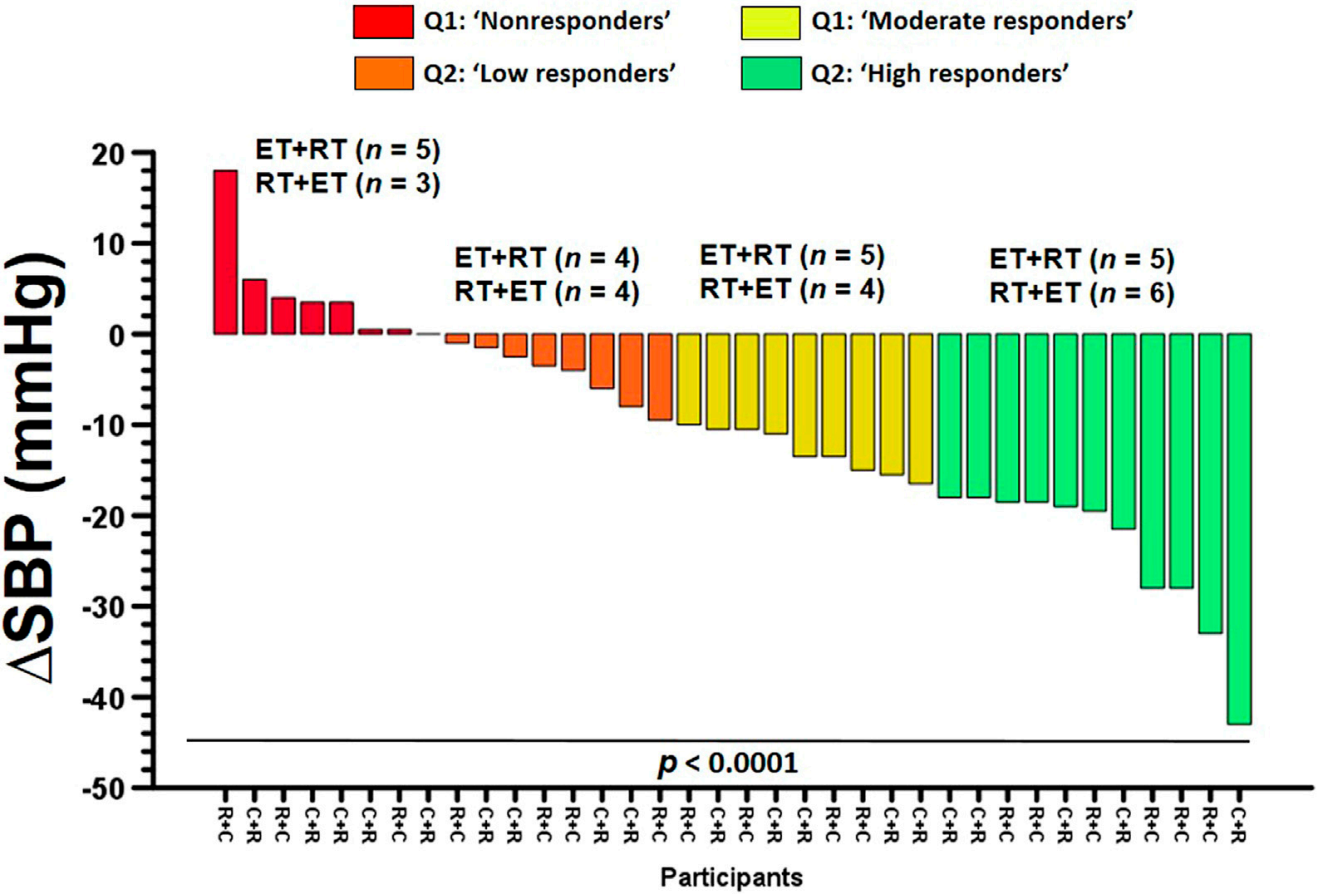
Interindividual response for systolic blood pressure participants of 4 weeks (eight exercise sessions) of two order groups of concurrent training in morbid obesity patients. Groups are described as (ET + RT) endurance training plus resistance training group and (RT + ET) resistance training plus endurance training group. Categories are described as (Q1: NRs) quartile 1nonresponders, (Q2: LRs) quartile 2low responders, (Q3: MRs) quartile 3moderate responders, and (Q4: HRs) quartile 4high responders to exercise training for decreasing blood pressure.

To the pre–post delta changes in ∆SBP, interindividual analyses revealed significant differences among quartile (Q) frequencies comparing Q1 “nonresponders” (*n* = 8; 22.2%), Q2 “low responders” (*n* = 8; 22.2%), Q3 “moderate responders” (*n* = 9; 25.0%), and Q4 “high responders” (*n* = 11; 30.5%), *p* < 0.0001 (Figure 6).

### Characterization of the exercise response to different concurrent training order

Quartile comparisons among anthropometric, body composition, cardiovascular, metabolic, dyslipidemia, endurance, and strength performance characteristics of “NRs” (Q1), “LRs” (Q2), “MRs” (Q3), and “HRs” (Q4) revealed significant differences in baseline SBP between Q4 vs. Q1, between Q4 vs. Q3, and between Q2 vs. Q1 (*p* = 0.035), (Table 2).

**TABLE 2:** Responders and nonresponders for post-exercise hypotensive effect by decreasing systolic blood pressure after 20 weeks of two orders of concurrent training.

	Q1: ‘NRs’	Q2: ‘LRs’	Q3: ‘MRs’	Q4: ‘HRs’	*p*-value†
	(SBP < −1 mmHg)	(SBP −1 to −9.9 mmHg)	(SBP −10 to −18 mmHg)	(SBP ≥ −18 mmHg)	
(n =)	8	8	9	11	
Age (y)	45.0 ± 11.3	40.0 ± 13.8	41.8 ± 11.7	47.0 ± 9.6	*p* = 0.776
Anthropometric					
Height (cm)	156.0 ± 3.5	160.1 ± 7.5	157.7 ± 9.7	157.7 ± 8.0	*p* = 0.837
Body mass (kg)	107.4 ± 33.8	106.3 ± 14.8	115.7 ± 19.5	101.2 ± 16.1	*p* = 0.844
Body mass index (kg/m^2^)	44.1 ± 7.9	41.5 ± 7.4	46.5 ± 4.6	40.7 ± 5.4	*p* = 0.242
Waist circumference (cm)	118.9 ± 21.8	112.0 ± 10.2	121.1 ± 11.7	111.5 ± 8.1	*p* = 0.376
Body composition					
Body fat (%)	47.1 ± 8.6	47.3 ± 3.4	49.2 ± 2.8	46.5 ± 4.6	*p* = 0.704
Body fat (kg)	52.7 ± 24.7	50.7 ± 9.9	57.2 ± 11.3	47.5 ± 11.0	*p* = 0.570
Lean mass (kg)	54.7 ± 9.7	55.7 ± 5.4	58.6 ± 9.5	53.7 ± 6.8	*p* = 0.635
Skeletal muscle mass (kg)	51.9 ± 9.2	52.9 ± 5.1	55.6 ± 9.1	51.0 ± 6.5	*p* = 0.634
Bone mass (kg)	2.7 ± 0.4	2.8 ± 0.2	2.9 ± 0.4	2.7 ± 0.3	*p* = 0.653
Total body water (%)	40.0 ± 8.3	40.7 ± 4.4	43.6 ± 8.7	39.2 ± 5.8	*p* = 0.602
BMR (kcal)	1737.8 ± 376.5	1764.6 ± 191.5	1867.6 ± 307.6	1683.2 ± 224.3	*p* = 0.576
Cardiovascular					
Systolic BP (mmHg)	122 ± 18	141 ± 9^Q1^	121 ± 16^Q2^	136 ± 17^Q1,Q3^	***p* = 0.035**
Diastolic BP (mmHg)	82.7 ± 12	93 ± 8	83 ± 11	87 ± 8	*p* = 0.173
Heart rate rest (beats/min)	81 ± 13	88 ± 9	77 ± 8	80 ± 12	*p* = 0.271
Pulse pressure (mmHg)					
Metabolic					
Diabetes					
Fasting glucose (mg/dl)	94.0 ± 11	96.0 ± 6.0	94.0 ± 5.0	91.0 ± 18.0	*p* = 0.968
Dyslipidemia					
Total cholesterol (mg/dl)	194.0 ± 14.0	159.0 ± 40.0	176.0 ± 26.0	173.0 ± 36.0	*p* = 0.545
LDL-c cholesterol (mg/dl)	120.0 ± 13.0	79.0 ± 37.0	106.0 ± 25.0	99.0 ± 32.0	*p* = 0.382
HDL-c cholesterol (mg/dl)	61.0 ± 15.0	39.0 ± 10.0	50.0 ± 12.0	51.0 ± 16.0	*p* = 0.366
Triglycerides (mg/dl)	114.0 ± 19.0	206.0 ± 37.0	120.0 ± 35.0	149.0 ± 125.0	*p* = 0.568
Endurance performance					
6Mwt (m)	541.2 ± 88.0	480.0 ± 176.6	510.0 ± 219.2	483.6 ± 105.2	*p* = 0.836
Strength performance					
Handgrip Nd (kg)	31.0 ± 15.3	31.2 ± 9.2	27.7 ± 6.3	29.1 ± 7.6	*p* = 0.877
Handgrip D (kg)	30.2 ± 16.5	30.5 ± 10.5	27.0 ± 5.5	28.5 ± 7.7	*p* = 0.893

Data are shown in mean and± standard deviation. Categories are described as (Q1: NRs) quartile 1nonresponders, (Q2: LRs) quartile 2low responders, (Q3: MRs) quartile 3moderate responders, and (Q4: HRs) quartile 4high responders to exercise training for decreasing blood pressure. Outcomes are described as (BMR) basal metabolic rate, (LDL-c) low-density lipids, (HDL-c) high-density lipids, (6Mwt) six-minute walking test, (Nd) non dominant hand, and (D) dominant hand. (†) Univariant test at *p* < 0.05.

### Predicting responders from baseline outcomes

After linear regression analyses, there were significant associations between the PEH outcome represented by the average of ∆SBP decreases (−4.7 mmHg) and the body fat percentage [β –3.826, *R* ^2^ 0.211 (21.1%), *p* = 0.031], skeletal muscle mass [β –2.150, *R* ^2^ 0.125 (12.5%), *p* = 0.023], fasting glucose [β 1.273, *R* ^2^ 0.078 (7.8%), *p* = 0.003], triglycerides [β 0.210, *R* ^2^ 0.014 (1.4%), *p* = 0.008], and 6Mwt [β 0.183, *R* ^2^ 0.038 (3.8%), *p* = 0.044] (Table 3).

**TABLE 3:** Association between the PEH represented by “SBP” decreases with anthropometric, body composition, cardiovascular, metabolic, dyslipidemia, endurance, and strength performance outcomes in morbid obesity patients’ participants of 20 weeks of two orders of concurrent training (ET + RT or RT + ET).

Predictors of the PEH by ‘SBP’ decreases	β	*R* ^2^ (% prediction SBP)	*p-*value
Age (y)	−0.001	0.000 (0.0%)	*p* = 0.997
Anthropometric			
Height (cm)	−0.373	0.053 (5.3%)	*p* = 0.222
Body mass (kg)	−0.050	0.007 (0.7%)	*p* = 0.655
Body mass index (kg/m^2^)	0.161	0.032 (3.2%)	*p* = 0.346
Waist circumference (cm)	1.107	0.002 (0.2%)	*p* = 0.057
Body composition			
Body fat (%)	−3.826	0.211 (**21.1%**)	***p* = 0.031**
Body fat (kg)	1.952	0.211 (21.1%)	*p* = 0.118
Lean mass (kg)	−0.198	0.016 (1.6%)	*p* = 0.499
Skeletal muscle mass (kg)	−2.150	0.125 (**12.5%**)	***p* = 0.023**
Bone mass (kg)	−3.768	0.014 (1.4%)	*p* = 0.537
Total body water (%)	−0.249	0.020 (2.0%)	*p* = 0.455
BMR (kcal)	−0.005	0.012 (55.7%)	*p* = 0.557
Cardiovascular			
Systolic BP (mmHg)	−1.166	0.125 (**12.5%**)	***p* = 0.023**
Diastolic BP (mmHg)	−0.211	0.030 (3.0%)	*p* = 0.309
Pulse pressure (mmHg)			
Heart rate rest (beats/min)	0.026	0.001 (0.1%)	*p* = 0.889
Metabolic			
Diabetes			
Fasting glucose (mg/dl)	1.273	0.078 (**7.8%**)	***p* = 0.003**
Dyslipidemia			
Total cholesterol (mg/dl)	0.159	0.113 (11.3%)	*p* = 0.187
LDL-c (mg/dl)	0.565	0.055 (5.5%)	*p* = 0.038
HDL-c (mg/dl)	0.198	0.045 (4.5%)	*p* = 0.416
Triglycerides (mg/dl)	0.210	0.014 (**1.4%**)	***p* = 0.008**
Endurance performance			
6Mwt (m)	0.183	0.038 (**3.8%**)	***p* = 0.044**
Strength performance			
Handgrip Nd (kg)	0.098	0.006 (0.6%)	*p* = 0.667
Handgrip D (kg)	0.057	0.002 (0.2%)	*p* = 0.794

Outcomes are described as (BMR) basal metabolic rate, (LDL-c) low-density lipid cholesterol, (HDL-c) high-density lipid cholesterol, (6Mwt) six-minute walking test), (Nd) non dominant hand, and (D) dominant hand. Bold values denote significant association by linear regression analyses tested at *p* < 0.05. Bold values also denote the % of prediction of the independent outcome to the systolic blood pressure target outcome.

## Discussion

The aims of this study were (1) to investigate the effect of CT order (ET + RT vs. RT + ET) on the blood pressure responses; 2) characterize these responses in responders and nonresponders, and 3) identify potential baseline outcomes for predicting blood pressure decreases as responders. The main findings of the present study were as follows: 1) both ET + RT and RT + ET promote PEH that is expressed in a similar ‘magnitude’ after eight CT acute exercise sessions [i.e., measured in four sessions (2, 4, 6, and 8 and into 4 weeks)] (Figure 3), 2) the PEH showed differences at the interindividual level (by quartile classification) in terms of prevalence for HRs, LRs, MRs, and NRs (Figure 6, and Table 2); and, finally, 3) baseline body composition [(i.e., body fat (21%), and skeletal muscle mass (12.%)], cardiovascular [i.e., SBP (12.5%)], metabolic [i.e., fasting glucose (7.8%), triglycerides (1.4%)], and physical fitness [i.e., 6Mwt (3.8%)] significantly predicted the PEH independent of the CT order (Table 3). Considering that literature has described relevant vascular benefits and mortality reduction by a minimum blood pressure decrease of −2 mmHg (Guimaraes et al., 2010), these results were displayed with clinically significant relevant effects considering the SBP decreases on average in a total of eight CT sessions measured in four opportunities in each ET + RT (∑∆SBP −5.7 mmHg) and RT + ET (−4.3 mmHg) (Figure 3), taking into account that both groups were classified at baseline as the HTN1 stage (Figures 2A,B).

As we observed similar PEH in both groups reported by the mean of the four measurements that we registered in eight exercise sessions during 4 weeks (ET + RT; ∑∆SBP −5.7 mmHg and RT + ET –4.3 mmHg), our findings are not minor. For example, a recent study developed in older adults of 60 years reported PEH monitoring 6 h after exercise cessation (by 24 h ambulatory blood pressure measurement). The authors showed significant decreases in SBP −13.5 and MAP −2.0 mmHg but not in ∆DBP (Cordeiro et al., 2018). In other reports, a reduction in ∆SBP of −8 mmHg was observed in adults after 2 h of ET (20 min cycling) (Chan and Burns, 2013), and other blood pressure decreases were observed after 70 min of exercise walking/cycling ∆SBP −16 and ∆DBP −8 mmHg (Macdonald et al., 2001). A study by Taylor-Tolbert et al. (2000), applying ET (45 min on a treadmill, at 70% of the maximum oxygen consumption), reported PEH decreases in ∆SBP −7.4 and ∆DBP −3.6 mmHg, where these values were observed to be maintained at 24 h post-exercise cessation. Another more recent study of two exercise protocols (i.e., RT at 40% and RT at 80% of intensity based on 1RM) measured the PEH effect from 5, 10, and 60 min after exercise cessation showed at only 10 min post-exercise similar reductions in ∆SBP and ∆DBP (−5 to −10 mmHg), being relevant that using RT, and independent of the exercise intensity, overweight women with HTN can obtain benefit from a few minutes of RT exercise (Cavalcante et al., 2015). Thus, the PEH can be promoted from a few min post-exercise, and this response could be maintained for several hours as a clinical benefit for HTN populations.

On the other hand, from our results using two different orders of CT in morbidly obese patients, we show in the present study during 4 weeks ∆SBP decreases of (session 2 −8.2 and −5.6 mmHg, session 4; −5.9 and −8.3, session 6; −3.7 and −1.4, and session 8; −1.3 and −3.4 mmHg, in both ET + RT and RT + ET, respectively), and in ∆DBP (session 2; −0.2 and −1.2, session 4; −3.3 and −1.1, session 6; −1.0 and −0.3, and session 8; +0.8 and −0.4 mmHg) in both ET + RT and RT + ET, respectively (Figure 3). Following this, and although the PEH phenomenon is not new, and as the ET has been the traditional exercise modality inducing blood pressure decreases at post-exercise, CT configurations using ET + RT or RT + ET order here in morbid obesity populations could also represent a similar or more important strategy for decreasing blood pressure and also other health-related outcomes when populations are suffering from multiple cardiometabolic risk factors as this morbid obesity sample.

Although we do not observe differences in the “magnitude” of the PEH in both main ∆SBP and ∆DBP, it was observed along the four measurements applied (during the eight CT sessions/during 4 weeks) that there was a significant trend for declining the ‘magnitude’ in the blood pressure decreases (Figure 3). In young normotensive adults after following two exercise protocols of moderate-intensity ET exercise (70% of the peak oxygen consumption, 30 min) or lower-intensity ET (40% of the peak oxygen consumption, 30 min), it was reported that the “magnitude” of the PEH effect was not intensity-dependent decreasing ∆SBP −5/∆DBP −1 mmHg registered 20 min after each moderate and higher exercise intensity (Simão et al., 2005). On the other hand, the “volume-dependence” in the PEH was also tested in previous reports. In this line, a study that tested two exercise protocols (normotensive and other at elevated/HTN1 blood pressure), applying three different exercise volumes (i.e., 15, 30, and 45 min of ET, at 70% of the peak oxygen consumption), and where the PEH was reported after 5, 10, 15, 30, 45, and 60 min post-exercise cessation, reported that no significant differences were detected for decreasing ∆SBP and ∆DBP until 60 min post-exercise cessation. The authors concluded that the minimum dose of ET (i.e., 15 min) at moderate intensity in these different volumes of exercise has potential benefits to be used as a non-pharmacological HTN treatment (Macdonald et al., 2000). Interestingly, previous studies have also added that the PEH can be promoted from a single exercise session, where the blood pressure decrease was highly related to the previous baseline blood pressure of the HTN patients (Ciolac et al., 2008). In other studies, independent of the “magnitude” and “volume” of the exercise session, the same authors have reported in HTN patients’ participants of 2 consecutively days of ET and high-intensity interval training (HIIT), both exercise modalities decreased blood pressure increasing the number of participants with blood pressure from HTN to normal categorization in the daytime (i.e.*,* ET from 42 to 61%; HIIT from 54 to 61%) (Ciolac et al., 2008).

Using a similar pre vs. post-exercise method for testing PEH and the same protocol of measuring post 10 min post-exercise cessation, Notarius et al. (2006) reported from pre to post-exercise cessation (i.e., a progressive volitional test cycling by ET) significant decreases in ∆SBP and ∆DBP (of –11 and –6 mmHg, respectively), to each outcome. However, the sample characteristics were not in a morbid obesity condition. Another study by Takahashi et al. (2000) showed that in young adults (22.4 years), after developing a brief exercise protocol (5 min of ET at 80% of the peak oxygen consumption), ∆SBP and ∆DBP decreases (of −2.3 and +1.7 mmHg, respectively), where, similarly, the sample size included normal weight participants. To the best of our knowledge, there is a scarcity of studies reporting NR prevalence and the characteristics of those NRs for decreasing SBP after exercise training, with our study having a particular novelty in this information.

On the other hand, the interindividual response to the PEH in terms of reporting the NR prevalence has been poorly studied than the blood pressure changes from continuous exercise training programs from middle (few weeks) and long-term (i.e., months) exercise duration. For example, starting with normotensive samples, 6 weeks of HIIT decreased ∆SBP −10.0 and ∆DBP −15.0 mmHg but also reported NRs to and DBP (72.7%), where both outcomes showed increased ∆SBP +10 and DBP +2.0. In this study, the authors reported that subjects with higher baseline blood pressure also reported higher blood pressure decreases (Higgins et al., 2015). In long-term exercise programs, after 24 weeks of ET (65–80% of peak oxygen consumption, by walking/jogging), strength (8–12 repetitions per set, eight exercises, 70–85% of 1RM), or CT, approximately ∼60% of subjects were NRs to a decrease in SBP/DBP. Interestingly, in this study of elevated BP/HTN1 characteristic, the authors reported that those Rs decreased ∆SBP −11.5, ∆DBP −9.8 mmHg, and NRs increased these outcomes (∆SBP +7.9, ∆DBP +4.9 mmHg) (Moker et al., 2014). As additional information, these authors also included 2-weeks of detraining, where this “detraining” response explained the 44.8% of the ∆SBP training response to ET, RT, and CT, and, more robustly, the DBP detraining response, baseline DBP, and the MetS risk factor load explained the 60.1% of the ∆DBP training response.

In terms of predicting exercise response, traditionally, when more baseline (i.e., subjects with diseases) outcomes of health-related parameters are shown by a participant, usually more exercise benefits are received after exercise. Thus, baseline conditions have been reported as an important exercise predictor (Álvarez et al., 2018). On the other hand, the ‘magnitude’ of the PEH effect has also been highly predicted 60 min after exercise (*R* ^2^: 0.77%), and until 24 h post-exercise cessation (*R* ^2^: 0.67%) future blood pressure decreases with exercise training (Hecksteden et al., 2013), as well as the detraining stage has also predicted blood pressure decreases (Moker et al., 2014). In the present study, we report in Q1 “NRs” (*n* = 8; 22.2%), being in other terms all the Q2 “LRs” (*n* = 8; 22.2%), Q3 ‘MRs’ (*n* = 9; 25.0%), and Q4 “HRs” (*n* = 11; 30.5%), in total (*n* = 28, 77.7%), participants that certainly respond to decrease SBP, but in different degrees. Following this, our linear regression analyses revealed that ∆SBP decreases were significantly predicted by outcomes 1) the body fat percentage (21.1%), skeletal muscle mass (12.5%), fasting glucose (7.8%), triglycerides (1.4%), and 6 Mwt (3.8%), where, from a healthy lifestyle perspective, it is very relevant for morbid obesity populations to consider the relevant increase in the skeletal muscle mass in order to ascertain their high impact on future body fat reduction in this disease.

Under a clinical approach, it is very relevant to increase the knowledge about the study of PEH due to the following reasons 1) PEH occurs immediately after exercise cessation (Fitzgerald, 1981), 2) PEH shows duration during 12–24 h and above in HTN populations, 3) subjects who experience PEH usually are not physically fit (Pescatello et al., 2007; Zaleski et al., 2019), and 4) inclusively, some authors report that PEH can be used as self-regulation strategy to increase exercise adherence (Zaleski et al., 2019). In this line, as both ET + RT and RT + ET order of concurrent training promote similar magnitude in PEH after 4 weeks (eight exercise sessions), our sample size at “elevated BP”/“HTN1 stage” and showing at 4 weeks to start session number 4 (see Figure 2) near the ‘normal BP’ classification, our results suggest that both ET + RT and RT + ET order of concurrent training can be used to reduce BP in morbid obesity populations at risk of MetS in order to avoid HTN. On the other hand, the prevalence of NRs by quartile categorization was 22.2% (*i.e.,* based on SBP chosen outcome), in which other LRs, MRs, and HRs (77.8% of the sample) elicited different SBP decreases (Figure 6). Thus, we encourage increasing the PEH study and including other more noble/technological equipment to explain other mechanisms involved. On the other hand, ∆SBP reductions of −2 mmHg have been related to functional and structural vascular improvements in endothelial markers, such as a decrease in pulse wave velocity of −0.54 m/s (Guimaraes et al., 2010). Additionally, a −2 mmHg in ∆SBP has also been related to a reduction in mortality from cerebral vascular accidents by 10% and from cardiovascular disease by 7% (Guimaraes et al., 2010). Thus, in the present study, NRs were considered for those participants who decreased a –1 mmHg in blood pressure. The mechanisms of how exercise decreases blood pressure could be potentially explained by 1) angiogenesis in the skeletal muscle (Fernandes et al., 2012), 2) a reduction of the peripheral vascular resistance (Correia et al., 2015), 3) a reduction of the arterial stiffness (Guimaraes et al., 2010), 4) improvements in the endothelial function (Ramirez-Jimenez et al., 2017), 5) an increase in production and action of nitric oxide levels (Izadi et al., 2018), 6) an increase in the major baroreflex control (Somers et al., 1991), and also by the 6) increased shear stress produced by exercise in the arterial wall (Hong et al., 2022), and, thus, a major arterial distensibility (Kobayashi et al., 2022).

Our study is not far from limitations, 1) we do not control the intervention from the diet patterns; however, we remember each session to not change the regular nutrition behaviour, and smoking, caffeine, and other sympathetic stimulants were strictly avoided and recorded for each participant, and 2) our PEH was immediately recorded after each CT group, and after 10 min of exercise at rest, where we do not extend measurements for more hours after the exercise, 3) we did not include a strict control group, 4) we do not control the air temperature and relative humidity of the exercise room, and 5) we recruited only one man by each group. Some strengths of our work were that 1) we studied a poorly known morbid obesity sample and 2) we used the frequently PEH reported method of (PEH = postexercise blood pressure—pre-exercise blood pressure) being highly comparable to these results, 3) NRs were determined by quartile, where the minimum clinically benefit was −2 mmHg following clinical benefits, and 4) we reported the PEH in patients with morbid obesity that have been poorly studied.

## Conclusion

The CT order of ET + RT and RT + ET promote a similar “magnitude” in the post-exercise hypotensive effects during eight sessions of both CT orders in 4 weeks of duration, revealing “nonresponders” and “high” responders that can be predicted from body composition and metabolic and physical condition outcomes.

## Novelty and significance

### What is new?

Independent of the concurrent training order (i.e., endurance plus resistance training or resistance plus endurance training), concurrent training promotes the post-exercise hypotension effect in morbid obesity conditions and a different interindividual response.

### What is relevant?

The postexercise hypotensive effect from both concurrent training orders promotes clinically blood pressure decreases, reducing the risk factors for metabolic syndrome and cardiovascular disease in morbid obesity patients and can be predicted from body fat and skeletal muscle mass baseline outcomes.

## Data Availability

The original contributions presented in the study are included in the article/Supplementary Material; further inquiries can be directed to the corresponding author.

## Supplementary material

Supplementary File S1 Study protocol.

## Abbreviations

CT, concurrent training; ET + RT, endurance plus resistance training; RT + ET, resistance training plus endurance training; PEH, post-exercise hypotension; HTN, arterial hypertension; IVET, interindividual variability to exercise training; BMI, body mass index; SBP, systolic blood pressure; DBP, diastolic blood pressure; MAP, mean arterial pressure; HR, heart rate at rest; PP, pulse pressure; NRs, nonresponders; LRs, low responders; MRs, moderate responders; HRs, high responders.
