# Supplementary figures and images for: Characterizing the interindividual postexercise hypotension response for two order groups of concurrent training in patients with morbid obesity
Source: Front Physiol. 2022 Oct 10;13:913645. doi: 10.3389/fphys.2022.913645 (PMC9589449; doi:10.3389/fphys.2022.913645)

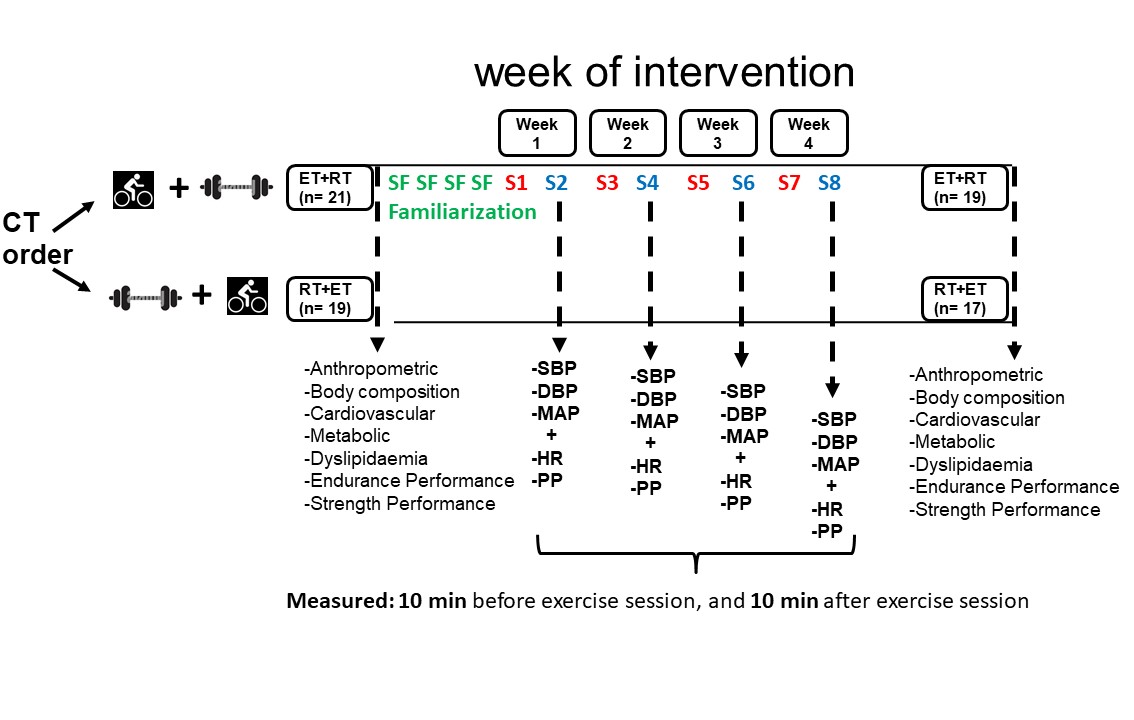

Supplement: Supplementary file 1 [file Image1.jpg]
